# The genetics of eye disorders in the dog

**DOI:** 10.1186/2052-6687-1-3

**Published:** 2014-04-16

**Authors:** Cathryn S Mellersh

**Affiliations:** Animal Health Trust, Lanwades Park, Kentford, Suffolk, CB8 7UU UK

## Abstract

Inherited forms of eye disease are arguably the best described and best characterized of all inherited diseases in the dog, at both the clinical and molecular level and at the time of writing 29 different mutations have been documented in the scientific literature that are associated with an inherited ocular disorder in the dog. The dog has already played an important role in the identification of genes that are important for ocular development and function as well as emerging therapies for inherited blindness in humans. Similarities in disease phenotype and eye structure and function between dog and man, together with the increasingly sophisticated genetic tools that are available for the dog, mean that the dog is likely to play an ever increasing role in both our understanding of the normal functioning of the eye and in our ability to treat inherited eye disorders. This review summarises the mutations that have been associated with inherited eye disorders in the dog.

**Electronic supplementary material:**

The online version of this article (doi:10.1186/2052-6687-1-3) contains supplementary material, which is available to authorized users.

## Lay summary

Inherited forms of eye disease are arguably the best described and best characterized of all inherited diseases in the dog, at both the clinical and molecular level and at the time of writing 29 different mutations have been documented in the scientific literature that are associated with an inherited ocular disorder in the dog. The dog has already played an important role in the identification of genes that are important for ocular development and function as well as emerging therapies for inherited blindness in humans. Similarities in disease phenotype and eye structure and function between dog and man, together with the increasingly sophisticated genetic tools that are available for the dog, mean that the dog is likely to play an ever increasing role in both our understanding of the normal functioning of the eye and in our ability to treat inherited eye disorders. This review summarises the mutations that have been associated with inherited eye disorders in the dog.

## Introduction

Inherited forms of eye disease are arguably the best described and best characterized of all inherited diseases in the dog, at both the clinical and molecular level. At the time of writing 29 different mutations have been documented in the scientific literature that are associated with an inherited ocular disorder in the dog (Table 1). Several more conditions have been described very well at the genetic and clinical level although their causal mutations remain elusive; however the genetic basis of many of these will undoubtedly be unraveled over the coming years, thanks to the increasingly sophisticated genetic resources that are now available for the dog.

**Table 1:** **Genes associated with inherited eye disorders in the domestic dog**

Disease	Locus or abbreviation	Gene	Breed	Reference
Cone-rod dystrophy	CRD3	*ADAM9*	Glen of Imaal terrier	[53, 54]
Primary open angle glaucoma	POAG	*ADAMTS10*	Beagle	[147]
Primary lens luxation	PLL	*ADAMTS17*	Multiple, mainly terrier breeds	[129, 132]
Rod cone degeneration	RCD4	*C2orf71*	Gordon Setter, Irish Setter, Tibetan Terrier	[30]
Generalised progressive retinal atrophy	gPRA	*CCDC66*	Schappendoes	[28]
Progressive retinal atrophy	PRA	*CNGB1*	Papillon	[15, 17]
Cone degeneration	CD	*CNGB3*	Alaskan malamute	[68]
Cone degeneration	CD	*CNGB3*	German shorthaired pointer	[69]
Dwarfism with retinal dysplasia (oculoskeletal dysplasia)	DRD2 (OSD2)	*COL9A2*	Samoyed	[90]
Dwarfism with retinal dysplasia (oculoskeletal dysplasia)	DRD1 (OSD1)	*COL9A3*	Labrador retriever	[90]
Hereditary cataract	HC, EHC	*HSF4*	Staffordshire bull terrier, Boston terrier, French bulldog	[103]
Hereditary cataract	HC	*HSF4*	Australian Shepherd	[107]
Collie eye anomaly	CEA	*NHEJ1*	Collies	[91]
Cone-rod dystrophy		*NPHP4*	Standard wirehaired dachshund	[49]
Photoreceptor dysplasia	PD	PDC	Miniature schnauzer	[13]
Rod cone dysplasia	RCD1	PD*E6B*	Irish setter	[2]
Rod cone dysplasia	RCD1	PD*E6B*	Sloughi	[3]
Rod cone dysplasia	RCD3	PD*E6A*	Cardigan Welsh corgi	[4]
Progressive rod-cone degeneration	PRCD	PRCD	Multiple breeds	[23]
Rod cone dysplasia	RCD2	*RD3*	Collie	[7]
Autosomal dominant progressive retinal atrophy	ADPRA	*RHO*	English mastiff	[24]
Congenital stationary night blindness	CSNB	*RPE65*	Briard	[58, 59]
X-linked progressive retinal atrophy	XLPRA2	*RPGR*	Mixed breed dogs	[18]
X-linked progressive retinal atrophy	XLPRA1	*RPGR*	Siberian Husky, Samoyed	[18]
Cone-rod dystrophy	CORD1 (CRD4)	*RPGRIP*	Dachshunds	[38]
Early retinal degeneration	ERD	*STK38L*	Norwegian elkhound	[11]
Canine multifocal retinopathy	CMR1	*VMD2/BEST1*	Great Pyrenees, English Mastiff, and Bullmastiff dogs	[74]
Canine multifocal retinopathy	CMR2	*VMD2/BEST1*	Coton de Tulears	[74]
Canine multifocal retinopathy	CMR3	*VMD2/BEST1*	Lapponian Herder	[75]

Why have so many inherited eye disorders been described in the dog? A principal reason is that the eye is very accessible, and much of it can be examined in detail using non-invasive techniques, making it relatively easy to detect abnormalities, even if they do not impair vision significantly. There are clinical screening schemes in place in many countries that offer dog breeders the opportunity to screen their dogs, ideally before they are bred from, for disorders known to be inherited in their breed. One such scheme is the British Veterinary Association/Kennel Club/International Sheep Dog Society Eye Scheme that operates in the United Kingdom ( http://www.bva.co.uk/canine_health_schemes/Eye_Scheme.aspx). This scheme covers 11 inherited eye disorders in over 50 breeds of dog. The European College of Veterinary Ophthalmologists (ECVO) Scheme ( http://www.ecvo.org/) is in use in seven European countries, and individual ECVO Diplomates work in accordance with the scheme in other countries to control presumed inherited diseases of the eye and its adnexa. In the United States the Orthopedic Foundation for Animals (OFA) and the American College of Veterinary Ophthalmologists (ACVO) maintain a joint Eye Certification Registry (ECR). OFA Eye Certification Registry exams are ophthalmic examinations, performed by ACVO Diplomates, to assess dogs for the presence or absence of observable hereditary ocular disease. Dogs with normal exam results receive OFA eye certification numbers valid for one year. The three schemes listed above, and other comparable schemes in place around the world, differ incrementally from one another in the precise ways in which they are operated, but they all serve to document and register dogs affected with, and free from, inherited eye diseases. Each dog that is clinically examined under any of these schemes receives a certificate on which the results of the examination are recorded and the findings are also recorded in the relevant registry/database, thus providing a wealth of data regarding the clinical characteristics and incidence of inherited eye disorders in different breeds of domestic dog.

## Review

### Diseases of the retina

**Figure 1:**
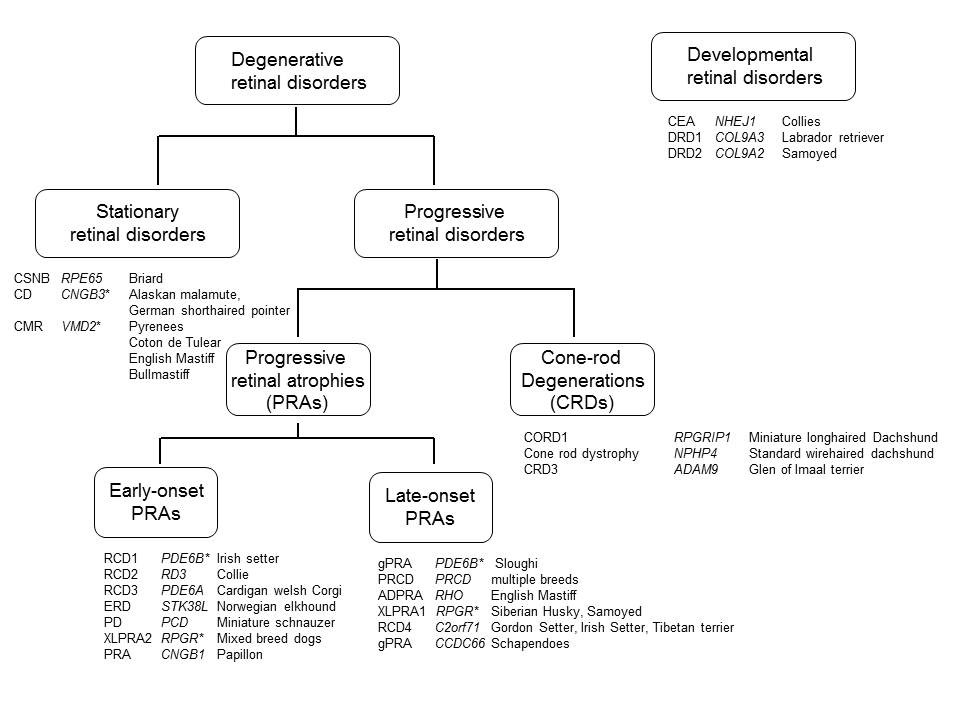
**Categorization of canine retinal disorders.** Different mutations in the genes marked with an asterix account for genetically distinct conditions.

Inherited forms of retinal disease are among the best clinically and genetically characterized genetic conditions in the dog. Retinal disorders can be categorized in various ways and the way in which they have been described in this review, which is summarized in Figure 1, is certainly not the only way to partition them. Most methods of classification will, however, broadly take into account the typical stage of development or age of onset of the disease, the cells that are typically affected and whether the disease becomes progressively severe during the dog’s lifetime or whether it is more or less stationary. Here the retinal disorders have been broadly divided into two main categories; the *degenerative* conditions where the retina develops normally and then degenerates during the dog’s lifetime and the developmental or *dysplastic* diseases in which the retina develops abnormally. It should be stressed, however, that categorising all retinal disease into these two broad forms is inevitably an over simplication in some cases, and that a minority of retinal diseases have both dysplastic and degenerative characteristics.

### Degenerative retinal disorders

The majority of retinal diseases that have been described in the dog are degenerative conditions. Some degenerative conditions are characterized by an inevitable increase in severity over time, invariably culminating in complete loss of vision, whereas other conditions are characterized by a pathology that does not deteriorate significantly throughout life. These two broad clinical categories of disease are described below under the headings *progressive* and *stationary* respectively.

#### Progressive retinal disorders

Progressive retinal atrophy (PRA) and cone-rod dystrophy (CRD) are collective terms for two broad forms of progressive, bilateral degenerative diseases that affect the retinal photoreceptor cells.

#### Progressive retinal atrophy

In general, PRAs are characterized by initial loss of rod photoreceptor function followed by that of the cones and for this reason night blindness is the first significant clinical sign for most dogs affected with PRA. Visual impairment in bright light invariably follows, accompanied by characteristic changes to the fundus that are visible upon ophthalmoscopic investigation. Typical changes include attenuation of the blood vessels of the retina, increased reflectivity of the tapetal layer as a result of retinal thinning and atrophy of the optic disc. In many dogs secondary cataracts develop, which might become extensive enough to obscure the retina and require the use of electroretinography (ERG) for diagnosis. Whereas most dogs show the same ophthalmoscopic abnormalities the age at which these abnormalities develop varies considerably between breeds and genetically different forms of PRA can be broadly divided into early- and late-onset forms.

##### Early-onset forms of PRA

Early onset forms of the disease are typically expressed between 2 and 6 weeks of age, the period of postnatal retinal differentiation in dogs, and are characterized by the abnormal development of the rod and cone photoreceptors. Four well-characterized, genetically distinct forms of autosomal recessive, early-onset retinal degeneration are rod-cone dysplasia type 1 (RCD1), rod-cone dysplasia type 2 (RCD2), rod-cone dysplasia type 3 (RCD3) and early retinal degeneration (ERD) [1]. RCD1, which affects Irish Setters from approximately 25 days after birth and culminates at about 1 year when the population of rods and cones is depleted, is caused by a nonsense mutation at codon 807 of the gene encoding the beta subunit of cGMP phosphodiesterase (PD*E6B*), an essential member of the phototransduction pathway [2]. This mutation was the first responsible for any form of PRA to be identified in the dog. An 8 base pair (bp) insertion after codon 816 in the same gene causes a genetically distinct form of PRA in the Sloughi which has a later age of onset than the Irish Setter form, with the first signs of visual impairment not being noticed until dogs are between 2 and 3 years of age [3]. PRA in the Cardigan Welsh corgi, termed rod-cone dysplasia 3 (RCD3), is also caused by a mutation in a subunit of cGMP phosphodiesterase, this time the alpha subunit, which results in a disease with a comparable age of onset to RCD1 [4]. In RCD3 affected dogs normal rod-mediated ERG responses fail to develop, photoreceptor outer segments do not reach maturity and rod cells are lost by apoptosis [5]. The genetically distinct RCD2 segregates in rough and smooth collies [6] and is caused by an insertion in *RD3* that results in a stretch of altered amino acids and an extended reading frame [7]. Mutations in *RD3* have been associated with retinal degeneration in both humans and mice [8].

Whereas the early onset forms of PRA, RCD1 and RCD3, described above, were among the first canine inherited diseases to be characterized at the molecular level, the mutation responsible for the similarly early onset condition ERD (early-onset degeneration) has only recently been identified. This condition, which was originally described in Norwegian Elkhounds [9], and was first mapped more than 10 years ago [10] is caused by an exonic SINE insertion in the gene *STK38L* [11]. Although known to have neuronal cell functions *STK38L* has not previously been associated with abnormal photoreceptor function; being associated with such a disease in dogs establishes this gene as a potential candidate for similar diseases in other species, including man.

A different form of early-onset PRA affects Miniature Schnauzers. Histologically this disease is evident from a very early age, when the normal retina is nearing the end of postnatal differentiation, and as it affects both rods and cones it is termed photoreceptor dysplasia (PD) [12]. This disease was originally associated with a missense mutation in phosducin (*PDC*) [13]. However additional research has since led to the complete exclusion of phosducin and to the identification of the gene and mutation that do in fact cause this disease, that is also known as Type A PRA [14]. Evidence suggests that Type A PRA is in fact a rare form of PRA in the Miniature Schnauzer and that other, genetically distinct forms of PRA segregate within the breed, for which the mutations have yet to be identified [14].

Recently a complex mutation, consisting of the combination of a one basepair deletion and a 6 basepair insertion was identified in exon 26 of *CNGB1* in Papillons with an early onset PRA. The mutation leads to a frameshift and a premature stop codon. Affected dogs demonstrated an early lack of rod function followed by a slow retinal degeneration, a phenotype comparable to mice and humans with *CNGB1* mutations [15]. *CNGB1* combines with *CNGA1* to form the rod cyclic nucleotide gated channel. Previous studies have shown the requirement of *CNGB1* for normal targeting of *CNGA1* to the rod outer segment [16] and indeed the authors were able to demonstrate a lack of detectable *CNGA1* protein in the rod outer segments of the affected Papillons homozygous for the mutation [15]. The same mutation was also described in Phalene dogs by Ahonen and collegues [17].

The early onset forms of PRA described above are all caused by mutations in autosomal genes. In contrast, a mutation in the X-linked retinitis pigmentosa GTPase regulator gene (*RPGR*) causes a very severe form of PRA, known as XLPRA2, that has been described in mixed breed dogs [18]. The XLPRA2 mutation is a 2 nucleotide deletion that results in a frameshift that significantly changes the predicted peptide sequence by leading to the replacement of many acidic glutamic acid residues with basic arginine residues and results in the premature termination of the protein 71 amino acids downstream. Unlike the genetically distinct, relatively late onset XLPRA1 that is described below, the phenotype associated with the frameshift mutation in XLPRA2 is very severe and manifests during retinal development. ERG abnormalities are evident by 5–6 weeks of age and cell degeneration is present by 4 months, suggesting the mutant protein has a toxic gain of function that severely compromises the early stage of development of the photoreceptors.

##### Late-onset forms of PRA

The late-onset forms of PRA are degenerations of photoreceptors that have completed normal development. Whereas the genes implicated in early-onset diseases are those necessary for the correct development of photoreceptors the genes associated with later-onset forms of disease are those necessary for the long-term maintenance and function of these cells.

Progressive rod cone degeneration (PRCD) is a late-onset form of PRA that affects multiple breeds. Prior to characterization of this disease at the molecular level, elegant interbreed crosses were undertaken to determine that the phenotypically similar diseases that were segregating in multiple breeds, including the miniature poodle, the English and American cocker spaniels, the Labrador retriever, the Australian cattle dog, the Nova Scotia duck tolling retriever and the Portugese water dog, were in fact allelic [19, 20]. However, when PRCD-affected dogs were mated to PRA-affected dogs of the Border Collie, Basenji and Italian greyhound breeds the progeny were normal, indicating these breeds are affected by genetically distinct forms of disease. The PRCD locus was mapped to a large region on CFA9 in 1998 [21] before the canine genome sequence was available and while tools with which to investigate the canine genome were relatively unsophisticated. However, the fact that a genetically identical disease segregated in so many breeds proved to be invaluable as it allowed the use of linkage disequilibrium mapping across affected breeds to considerably narrow the PRCD-associated region [22]. This led to the eventual identification of a single nucleotide substitution in the second codon of a previously unknown gene that is now known to be the cause of PRCD in at least 18 different breeds [23]. Intriguingly, an identical homozygous mutation was identified in a human patient with recessive retinitis pigmentosa, the human equivalent of PRA, and established the novel retinal gene, *PRCD*, as an important gene for the maintenance of rod photoreceptor structure and function across species.

A genetically distinct, late onset PRA has been described in the English Mastiff. This disease is unique, to date, among canine inherited retinopathies in that it is inherited as an autosomal dominant disease, and is caused by a single non-synonymous C → G transversion at nucleotide 11 of rhodopsin (*RHO*) that changes Thr-4 to Arg (T4R). Dogs carrying the *RHO* mutation have normal photoreceptor-specific ERG function at 3 to 6 months of age but by 13 months these responses are abnormal. In young affected dogs retinal structure, rhodopsin expression and photoreceptor activation is normal; disease progression is characterized by regions of initial focal photoreceptor degeneration surrounded by areas of structurally normal retina, which interestingly is very similar to the phenotypes of humans with *RHO* mutations [24]. A characteristic component of the phenotype associated with T4R mutation is the dose–response relationship that has been demonstrated between light exposure and the early alterations in retinal tissue that occur in affected animals. Highest doses of light cause rapid loss of neurons, reaching complete degeneration of photoreceptors in < 4 weeks whereas the lowest doses of light exposure enable mechanisms acting over a time scale of weeks to months to repair the abnormal alterations resulting from neuronal stress [25]. This mutation, originally identified in the English mastiff, has also been identified in PRA-affected bull mastiffs but has not been identified in any other breeds to date [26].

A different late onset form of autosomal recessive generalised PRA has been described in Schapendoes where the age of onset is typically between 2–5 years. During the early stages of the disease affected dogs become night-blind, lacking the ability to adjust their vision to dim light; later their daytime vision also fails. This process of complete photoreceptor degeneration takes up to 2 years [27]. The causal mutation for the disease has been shown to be a single bp insertion in exon 6 of the recently discovered gene coiled-coil domain containing 66 (*CCDC66*) that leads to a stop codon. *CCDC66* is evolutionarily conserved in different vertebrate species and exhibits a complex pattern of differential RNA splicing resulting in various isoforms in the retina. Immunohistochemically, CCDC66 protein is detected mainly in the inner segments of photoreceptors in mouse, dog, and man although the retinas of affected Schapendoes have been shown to lack CCDC66 protein [28].

A different mutation in *RPGR* from that associated with XLPRA2 (described above) is responsible for a sex-linked form of late-onset form PRA that was originally described in the Siberian Husky [29] known as XLPRA1. The mutation, which has also been identified in the Samoyed, is a five nucleotide deletion that causes a frameshift and an immediate premature stop; the truncated protein lacks 230C-terminal amino acids which causes a slight decrease in the isoelectric point [18]. The photoreceptors of dogs that carry this mutation develop normally, in contrast to those of dogs with XLPRA2, and remain morphologically and functionally normal until young adulthood, indicating the C-terminal of the *RPGR* protein is not essential for functional and structural differentiation of rods and cones.

Recently a frameshift mutation was identified in *C2orf71* that causes an autosomal recessive form of late onset PRA in the Gordon and Irish Setters [30]. The average age of onset in the dogs studied was approximately 10 years of age. This variant was homozygous in 19 of 21 PRA cases and was at a frequency of approximately 0.37 in the Gordon Setter population. Approximately 10% of cases in this study (2 of 21) were not associated with the *C2orf71* mutation, indicating that PRA in this breed is genetically heterogeneous and caused by at least two mutations. This variant is also present in a number of Irish Setter dogs with PRA and has an estimated allele frequency of 0.26 in the breed. The function of *C2orf71* remains unknown, but it is important for retinal development and function and has previously been associated with autosomal recessive retinitis pigmentosa in humans [31–34]. The form of PRA associated with the mutation in *C2orf71* has been termed RCD4, for rod-cone degeneration 4, to distinguish it from other forms of rod-cone degeneration [30]. The mutation has also been found in Tibetan Terriers affected with PRA (Mellersh and Downs, unpublished).

All the progressive, late-onset retinal disorders described behave, more or less, as single-gene conditions, caused by highly penetrant mutations. There is, however, some evidence that environmental modifiers may play a role in some of these diseases, causing phenotypic variation between and within breeds [20].

#### Cone-rod degenerations

Cone-rod dystrophies are disorders predominantly of cones, with rods becoming affected later. CRDs have ophthalmoscopic changes that are very similar to those of PRA and detailed ERG studies that measure both cone and rod-specific responses are required to distinguish between the two types of condition. For this reason several disorders have been initially described as PRAs to be later re-classified when extensive ERG investigations have been undertaken.

One such disorder is a form of retinal degeneration that has been described in the Miniature longhaired dachshund (MLHD). The disease was originally described as an early-onset, autosomal recessive PRA with all affected dogs within an inbred research colony displaying ophthalmologic abnormalities that were detectable by ERG by six weeks of age and 25 weeks by fundoscopy and becoming blind by the time they were 2 years of age [35]. A subsequent electroretinography study identified an initial reduction of the cone photoreceptor function which led to the condition being re-classified as a cone-rod dystrophy (CRD), rather than a rod-led PRA, and the disease was termed CORD1 for cone-rod degeneration 1 [36]. The same condition has also been referred to as *CRD4* by others, for cone-rod degeneration 4 [20]. Later findings by Lheriteau and co-workers were also consistent with the condition being a CRD [37]. Using the same colony of dogs CORD1 was mapped to a large region on CFA15 and a mutation in *RPGRIP1* was identified that co-segregated completely with CORD1 in the research colony [38]. The mutation is a 44 bp insertion of a A29 tract flanked by a 15 bp duplication in exon 2 of the gene, that creates a frameshift and introduces a premature stop codon early in exon 3. Mutations in *RPGRIP1* have been associated with Leber congenital amaurosis (LCA) [39], retinitis pigmentosa (RP) [40] and CRD [41] in humans, as well as inherited retinal abnormalities in mice [42] which suggests it plays an important role in visual function. The gene product’s precise role is not currently understood but it is thought to anchor regulatory complexes at the photoreceptor connecting cilium, which acts as a bridge between the inner and outer segments of photoreceptor cells [43] as well as having functions in disk morphogenesis [42] and in the structure of the ciliary axoneme [44]. *RPGRIP1* also interacts with *NPHP4*, a gene that has been associated with a genetically distinct form of early-onset CRD segregating in the standard wire-haired variety of Dachshund [45–49]. Within the research colony of MLHDs there was complete correlation between the *RPGRIP1* genotype and phenotype of the dogs with respect to their CORD1 phenotype whereas in the pet MLHD population this was not the case [50]. Outside of the colony there was considerable variation in the age of onset of retinal degeneration in dogs that were homozygous for the *RPGRIP1* insertion (termed *RPGRIP1*-mutant), which has also been identified in other breeds, including the English springer spaniel (ESS) and the Beagle. In a study of a small number of *RPGRIP1*-mutant Beagles ERG cone responses were undetectable whereas rod responses were variable between dogs, and between eyes of the same dog [50]. In the same study all *RPGRIP1*-mutant MLHDs showed reduced cone responses, even in the absence of ophthalmoscopic abnormalities, a finding that has also been corroborated by Busse and co-workers [51]. Together these findings suggest that additional mutations are involved which modify the age of onset of ophthalmoscopic abnormalities associated with the *RPGRIP1* mutation. Because the original research colony used was developed from a very small number of dogs it is a real possibility that the colony was fixed for these additional loci which, therefore, went undetected until the more outbred pet population was investigated. The mutation in *NPHP4* described above, that causes an early onset cone-rod dystrophy in standard wire-haired dachshunds [49] was not present in the dachshunds studies by Miyadera, enabling that mutation to be excluded. A recent association study using *RPGRIP1*-mutant MLHDs that had either early or late onset cord1 has indeed revealed a second locus that segregates with early-onset disease [52], indicating early onset CRD in MLHDs is more likely to be a digenic condition, and that the *RPGRIP1* insertion alone causes a late onset CRD, although ERG abnormalities may be detected early in life.

Another form of canine cone-rod dystrophy to be characterized at the molecular level is crd3, for cone-rod dystrophy 3, that segregates in the Glen of Imaal terrier. This disease becomes evident ophthalmoscopically in affected dogs as young as 3 years of age, and progresses to end-stage retinal degeneration over several years. Very recently the causal mutation has been identified by two research groups almost simultaneously, as a large genomic deletion of *ADAM9* (A Disintegrin And Metalloprotease domain, family member 9) that removes exons 15 and 16 of the *ADAM9* transcript [53, 54] and generates a premature stop codon that is predicted to result in a truncated protein that lacks critical domains. This finding established *CRD3* as a true orthologue, and a potentially useful model, of the similar human condition CORD9 in which four distinct *ADAM9* mutations have been found [55].

#### Stationary retinal disorders

The forms of both PRA and CRD described above are all inherited retinopathies that are characterized by increasing severity and decreasing visual function over time. Progressive retinal changes during the dog’s lifetime invariably lead to complete blindness.

The first non-progressive retinopathy to be well-characterized was described in the Swedish Briard by Narfstrom and colleagues [56] as stationary and congenital, resulting in it being termed congenital stationary night blindness (CSNB). Since the initial report the disease has also been described as having a progressive component leading to it also being called a hereditary retinal dystrophy [57]. However CSNB and hereditary retinal dystrophy have since both been shown to be caused by a four nucleotide deletion in exon 5 of the *RPE65* gene, indicating they are genetically identical conditions [58, 59]. *RPE65* is involved in the conversion of all-*trans*-retinoids to 11-*cis*-retinoids and in its absence the visual cycle is interrupted, resulting in a lack of visual pigment [60]. This canine disease has a very characteristic clinical phenotype; affected dogs have profound visual impairment present from at least 5–6 weeks of age, but remain ophthalmoscopically normal, at least for the first 3–4 years of life. Older dogs may show subtle retinal abnormalities indicative of a slowly progressive retinal degenerative process. Both cone and rod mediated ERG responses are highly abnormal, probably due to a combination of responses from rods and possibly cones with very reduced sensitivity [58]. It was the unique absence of visual function in dogs with healthy rod photoreceptors that was observed in CSNB-affected dogs that led to landmark studies in the field of retinal gene therapy. Subretinal injections of adeno-associated virus vectors expressing *RPE65* resulted in restoration of rod photoreceptor function and improved visual function, first in dogs [61, 62] and subsequently in humans [63–65].

Cone degeneration (CD) is also different from other progressive disorders in that early-onset cone degeneration occurs in the absence of the subsequent rod degeneration that characterizes cone-rod dystrophies. In *cd*, which was originally described in Alaskan Malamutes [66], affected puppies develop day-blindness and photophobia between 8 and 12 weeks of age, when retinal development is normally completed in dogs, although these clinical signs only occur in bright light and the dogs remain ophthalmoscopically normal throughout their entire lives. Cone function starts to deteriorate by the age of 6 – 12 weeks and is unrecordable in adult dogs [67]. Rod photoreceptors, however, remain functionally and structurally normal throughout the animal’s life. A large genomic deletion that removes all exons of *CNGB3*, the gene that encodes the β subunit of the cone cyclic nucleotide-gated cation channel, has been identified in CD-affected Alaskan Malamute-derived dogs, although there is evidence that the condition might be genetically heterogeneous in this breed as some dogs have been identified with clinical signs of day blindness that lack the *CNGB3* deletion [68]. A missense mutation in the same gene has been detected in German Shorthaired Pointers affected with a clinically identical allelic disorder [69]. These findings established *CD* as an orthologue of human achromatopsia, a condition also known as rod monochromacy or total congenital colour blindness, that shares many of its clinical features with CD and has also been associated with mutations in *CNGB3* [70, 71]. The potential of these orthologues has recently been demonstrated by the successful restoration of cone function and associated photopic vision in both of the canine achromatopsia models by gene replacement therapy [72].

Another inherited retinal disorder that is generally non-progressive is canine multifocal retinopathy (*CMR*), a disease that has been recognized in several breeds, particularly Great Pyrenees, Coton de Tulear, English Mastiff and Bullmastiff [73, 74]. Ophthalmoscopic changes are usually evident in affected dogs before the age of around 4 months and are characterized by multifocal areas of retinal elevation that contain subretinal accumulation of serous fluid. Retinal elevations can remain static for several years, whereas multifocal outer retinal atrophy is often seen in older animals. Several different variants in the Bestrophin gene (*BEST1* (alias *VMD2*)) have been identified as likely causal mutations for *CMR* in the dog. In Great Pyrenees, English Mastiff, and bullmastiff dogs, a C73T mutation in exon 2 causes a premature translation termination that limits the open-reading frame to 25 codons, compared with 580 codons in the wild-type mRNA (cmr1) and in Coton de Tulears a G482A transition changes an evolutionarily conserved glycine residue to aspartic acid (cmr2). In Lapponian Herders two coding changes have been described in CMR affected dogs; a deletion at nucleotide position 1,388 (c1388del) and a substitution at nucleotide position 1,466. The c1388del results in a frame shift (Pro463*fs*) introducing a new stop codon at amino acid 490 and the G1466T substitution by itself leads to a conservative change in the amino acid sequence (Gly489Val), which is predicted to change the protein function with only marginal significance. In combination with the C1388del, however, the G1466T substitutions results in an additional stop codon at amino acid position 489 within the shifted reading frame (Gly489X). Since the mutations have only been found in complete linkage disequilibrium, the authors conclude that the combination of changes results in the disease they refer to as *cmr3* [75].

These mutations establish *CMR* as a novel animal model for Best macular dystrophy (BMD) in humans, an autosomal dominant, childhood retinal disease also caused by mutations in the Bestrophin gene [76, 77].

#### Developmental diseases

Retinal dysplasia is the term used to denote disorderly proliferation and imperfect differentiation of the developing retina and can be subdivided into focal, multifocal, geographic and total types. Focal and multifocal types manifest as linear folds and ‘rosettes’ of tissue in the inner (sensory) retinal layer whereas in geographic forms there are larger areas of defective retinal development that appear as large irregular or horseshoe-shaped areas of mixed hyper- or hyporeflectivity in the central retina. Total or generalized forms of retinal dysplasia have been described as an inherited trait in several breeds, including the Bedlington terrier [78], Sealyham terrier [79], Labrador retriever [80] and the Yorkshire terrier [81] and are associated with complete detachment of the abnormal neuroretina from the retinal pigment epithelium that results in blindness of affected eyes. All forms of retinal dysplasia are congenital and non-progressive. Retinal dysplasia appears to be inherited as an autosomal trait, at least in those breeds where sufficient numbers of individuals have been studied to reliably estimate the mode of inheritance [82–84]. The genetics of isolated or non-syndromic forms of retinal dysplasia have not been characterized at the molecular level in any breeds to date and no mutations have been associated with this condition.

Forms of syndromic retinal dysplasia have been reported in the Labrador retriever [85–87] and the Samoyed [88]. Homozygous affected dogs had short-limbed dwarfism and a range of ocular changes characterized by complete retinal detachment and cataract whereas heterozygous dogs had only focal or multifocal retinal lesions [85, 86]. Breeding studies determined that these two disorders are non-allelic [89] and they were termed DRD1 (dwarfism with retinal dysplasia type 1, Labrador retriever) and DRD2 (Samoyed), respectively (these conditions have also previously been referred to as *OSD1* and *OSD2* for oculoskeletal dysplasia). Mutations have recently been associated with both disorders; a 1-base pair insertional mutation in exon 1 of *COL9A3* is associated with DRD1 and a 1,267-bp deletion in the 5’ end of *COL9A2* co segregates with DRD2. Both mutations affect the COL3 domain of their respective genes, the expression of which are both reduced in affected retinas [90].

Another complex congenital defect of the retina is collie eye anomaly (CEA), although retinal involvement is secondary to the primary ocular defects associated with this disorder. The primary phenotypic element of the disorder is regional hypoplasia of the choroid, the highly vascular layer underlying the retina. Associated retinal lesions, known as colobomas are often detectable ophthalmoscopically, as are tortuous retinal vessels and multiple retinal folds in a minority of cases [91]. CEA, which segregates in several herding breeds with Collie ancestry, was mapped to a large region of CFA37 that included over 40 genes [92]; subsequently the fact that the disorder segregates in multiple, closely related breeds was used to reduce the size of the critical disease-associated region and pinpoint the causal mutation to a 7.8 kb intronic deletion in the *NHEJ1* gene, which spans a highly conserved binding domain to which several developmentally important genes bind [91]. The precise mechanism by which the deletion causes CEA has not however been established to date.

### Hereditary cataract

The lens is the transparent, biconvex, avascular structure in the anterior segment of the eye that is partly responsible for the refraction of light to be focused on the retina. The lens consists of a nucleus, cortex and capsule and is suspended by many dense zonular ligaments which are attached to the capsule and connect between the ciliary body and the lens equator. Transparency is a crucial property of the lens which is achieved, in part, by the absence of light-scattering organelles within the lens fibres. New lens fibres are generated from the equatorial cells of the lens epithelium, which elongate, synthesize crystallin and finally lose their nuclei as they become mature lens fibres. The crystallins, which make up over 90% of the proteins in the lens, are specially adapted to contribute to the maintenance of transparency by forming soluble, high-molecular weight aggregates that need to stay in solution for the duration of an individual’s life.

Cataracts are simply defined as opacities of the lens and can develop for a variety of reasons, including advanced age and the secondary effects of other diseases such as diabetes or progressive retinal atrophy, and trauma. Primary or hereditary cataracts (HC) are common among dogs and are a leading cause of blindness. HC has been reported in as many as 97 different breeds [93, 94], with around 60 breeds being reported to be at increased risk compared to mixed-breed dogs [95]. Hereditary cataracts reported in different breeds vary with respect to their anatomic position within the lens, their age of onset and their progressive or stationary nature, although within a breed cataracts usually display marked breed specificity. Despite the large number of breeds affected by HC only a single gene, the transcription factor *HSF4*, has been implicated in the development of cataracts in dogs to date. *HSF4* belongs to a family of heat shock transcription factors that regulate the expression of heat shock proteins in response to different stresses, such as oxidants, heavy metals, elevated temperatures and bacterial and viral infections [96]. Different mutations in *HSF4* have been reported to cause both human autosomal dominant and recessive cataracts [97–99] and studies in mice have shown *HSF4* is required for normal fibre cell differentiation during lens development [100, 101]. Disruption of the gene leads to the development of cataracts via multiple pathways, including the down-regulation or loss of post-translational modification of different crystallin proteins [102]. A single recessive nucleotide insertion in exon 10 of the gene (CFA5 g.85286582_85286583insC), that causes a frameshift and introduces a premature stop codon, is responsible for an early onset, bilaterally symmetrical and progressive form of HC in the Staffordshire bull terrier [103]. This cataract starts to develop from a few months of age and invariably progresses to total cataract within 2–3 years if left untreated [104]. The mutation is shared by the Boston terrier, in which it causes the clinically identical early-onset hereditary cataract (EHC), one of two genetically distinct forms of cataract known to affect this breed [104, 105]. The mutation associated with the clinically more variable, late-onset hereditary cataract (LHC) in this breed has yet to be identified [106]. The same mutation has also been identified in a small number of French bulldogs with a clinically identical cataract (Mellersh, unpublished).

A single nucleotide deletion at the same position in *HSF4* (CFA5 g.85286582delC) has also been associated with HC in the Australian Shepherd. The form of cataract caused by the insertion identified in the Staffordshire bull terrier and related breeds has a recessive and highly penetrant mode of inheritance, is early onset, highly progressive and uniform. In contrast, the form of cataract observed in the Australian Shepherd, caused by the deletion described above, has a dominant, or co-dominant mode of inheritance, is not completely penetrant and is typically associated with a posterior polar subcapsular cataract that also has a variable age of onset. It is highly likely that other mutations associated with the development of cataracts are co-segregating in the Australian Shepherd population because not all the dogs with bilateral posterior polar subcapsular cataract carried a copy of the *HSF4* deletion [107].

*HSF4* has been excluded from involvement in the development of HC in a long list of breeds, including the Alaskan Malamute, American Cocker spaniel, Bichon Havanais, Belgian Shepherd Tervueren and Groenendael, Dachshunds, English Cocker spaniels, English Miniature Terrier, Finnish Lapphund, Golden retriever, Griffon Bruxellois, Kromfohrlander, Jack Russell terrier, Lapponian Herder, Miniature Schnauzer, Miniature Pinscher, Nova Scotia Duck Tolling Retriever, Rottweiler, Samoyed, Schnauzer, and Tibetan Mastiff [103, 107–111]. The paucity of canine cataract mutations that have been reported in the literature, compared to those associated with, for example, inherited retinal degenerations in the dog, is testament to the fact that HC is probably a genetically complex disorder in most breeds of dog and studies to date have not included the analysis of sufficient numbers of cases and controls to identify DNA variants associated with the disease. A recessive mode of inheritance has been suggested for congenital cataracts and microphthalmia in the Miniature Schnauzer [112] as well as cataracts in the Entlebucher mountain dog [113], the Bichon Frise [114] and the American Cocker spaniel [115]. In contrast, an autosomal dominant mode of inheritance with a high degree of penetrance has been suggested for the pulverulent (dust-like) form of cataract observed in the Norwegian Buhund [116] and autosomal dominant with variable penetrance has been suggested for inherited posterior polar subcapsular cataracts in the Labrador and Golden retriever [117], although current anecdotal evidence indicates that in the Labrador cataracts could also be inherited as an autosomal recessive trait. Evidence of inheritance has been reported for a handful of other breeds, including the Leonberger, Jack Russell terrier and Chow chow, although the precise mode of inheritance has rarely been identified [111, 118, 119].

### Primary lens luxation

Primary lens luxation (PLL) is not a disease of the lens itself, but rather an inherited deterioration of the lens suspensory apparatus, the zonule, which is a system of fibres that suspend the lens from the ciliary body, maintaining it within the visual axis and in contact with the anterior surface of the vitreous body. In dogs affected with PLL ultrastructural abnormalities of the zonular fibers are already evident at 20 months of age [120] long before the lens luxation that typically occurs when the dogs are 3 to 8 years old, as a result of degeneration and breakdown of the zonules which cause the lens to be displaced from its normal position within the eye [121–124]. In the majority of cases the dislocated lens will pass into the anterior chamber where its presence is likely to cause acute glaucoma. The condition has been recognized as a canine familial disorder for more than 100 years [125, 126] and is encountered at high frequency in several terrier breeds and in some other breeds with probable terrier co-ancestry [121–124, 127]. PLL is recessively inherited in the Tibetan terrier [127] and inheritance has been suggested to be recessive in the Shar Pei and other Western terrier breeds in which it has been studied [128]. A mutation in *ADAMTS17* has been described as the cause of PLL in three breeds, the Miniature Bull terrier, the Lancashire Heeler and the Jack Russell terrier. The mutation is a G→A substitution at c.1473 + 1, which destroys a splice donor recognition site in intron 10 and causes exon skipping that results in a frameshift and the introduction of a premature termination codon [129]. The great majority of PLL-affected dogs are homozygous for the mutation, but a small minority are heterozygous, leading to speculation that carriers, of some breeds at least, might be at increased risk of developing the condition compared to dogs that are homozygous for the wildtype allele [129]. *ADAMTS17* is one of 29 known mammalian members of the ADAMTS family of genes that encode secreted metalloproteases that proteolytically modify extracellular structural proteins. Mutations in a variety of *ADAMTS* genes have been associated with a diverse set of human diseases including Ehlers-Danlos syndrome [130] and Weill-Marchesani syndrome [131]. The canine *ADAMTS17* splice site mutation is shared by at least 17 different breeds, many of which are terriers or terrier-type breeds, but some of which have more diverse origins [132]. Some breeds that are known to be at increased risk of PLL, such as the Border Collie, do not carry the same *ADAMTS17* mutation as the terrier breeds, indicating their form of the disease must be genetically distinct although clinically similar [132].

### Other conditions

The diseases of the lens and retina described above represent the overwhelming majority of inherited eye conditions in the dog for which causal mutations have been identified. Many other ocular conditions have been reported to be more common in certain breeds than others, which is indicative that they have a genetic component. However, a rigorous estimate of the mode of inheritance has been undertaken for relatively few of these conditions. To list comprehensively all the eye conditions that have been reported in dogs is outside the scope of this review, so the remainder of conditions described is restricted to those conditions for which an estimate of the mode of inheritance or the heritability has been reported.

#### Glaucoma

Glaucoma is the term used to describe a group of conditions that result in increased intraocular pressure, with damage to the retinal ganglion cells and their axons, leading to vision loss and blindness. Glaucoma is commonly divided into congenital, primary and secondary types, depending on the aetiology of the condition. Congenital glaucoma is rare in the dog [133] and secondary glaucoma, which is the most common form of the condition observed in the dog, arises as result of antecedent or concurrent ocular disease, so is not itself inherited, although the primary, causal condition might be. Primary glaucoma occurs in the absence of any other ocular disease, and, therefore, is presumed to have a genetic component in most breeds. Primary glaucoma can occur in the presence (angle closure glaucoma) or absence (open angle glaucoma) of an abnormal, narrowed or closed opening into the ciliary cleft, which prevents the efficient drainage of aqueous humour from the anterior chamber of the eye, via the iridocorneal angle through openings between the pectinate fibres. Goniodysgenesis is the most common cause of primary glaucoma in dogs, and refers to the presence of abnormal, irregularly-shaped or imperforate sheets of pectinate fibres. Glaucoma has been reported to be more prevalent than average in several breeds, including the Flat Coated Retriever, American Cocker spaniel, the Bassett Hound, the Shar Pei, the Norwegian Elkhound and the Boston terrier [134–137]. A strong and significant correlation between goniodysgenesis and glaucoma was reported in the Great Dane, and the same study reported a high heritability for goniodysgenesis, suggesting glaucoma may be heritable in this breed [138]. A similarly significant association has been reported between pectinate ligament dysplasia and adult-onset primary glaucoma in the Flatcoated retriever, for which the heritability was estimated to be approximately 0.7 [139, 140]. To date no mutations have been identified that are associated with angle closure glaucoma in any breed of dog although the first glaucoma-associated locus has recently been identified in Dandie Dinmont Terriers [141].

Autosomal recessive, primary open-angle glaucoma (POAG) has been very well characterized in the Beagle [142–146] and a Gly661Arg variant in *ADAMTS10* has been associated with the condition in Beagles that developed elevated intraocular pressure from 8 to 16 months of age, due to increased resistance to outflow of aqueous humour despite normal appearing open iridocorneal angles [147].

#### Persistent hyperplastic primary vitreous

Persistent hyperplastic primary vitreous (PHPV) is a congenital, non-progressive condition which results from the abnormal regression of the foetal hyaloid vasculature. The condition is rare but is seen more commonly in Staffordshire bull terriers in which pedigree analysis supports a hereditary etiology for the condition but is insufficient to determine the exact mode of inheritance, [148, 149]. PHPV and persistent hyperplastic tunica vasculosa lentis (PHTVL) has also been described in detail in the Doberman [150].

## Conclusion

At the time of writing 29 different mutations have been associated with inherited eye disease in the domestic dog, and more are likely to have been identified by the time this review goes to press. This number far exceeds those associated with any other category of disease, meaning that inherited eye diseases are arguably better understood, at both the clinical and genetic level, than any other category of canine disease. The dog has already played an important role in emerging therapies for inherited blindness in humans and similarities in disease phenotype and eye structure and function between dog and man, together with the increasingly sophisticated genetic tools that are available for the dog, mean that the dog is likely to play an ever increasing role in both our understanding of the normal functioning of the eye and in our ability to treat inherited eye disorders.

## Authors’ original submitted files for images

Below are the links to the authors’ original submitted files for images. Authors’ original file for figure 1
