# Supplementary figures and images for: The genetics of eye disorders in the dog
Source: Canine Genet Epidemiol. 2014 Apr 16;1:3. doi: 10.1186/2052-6687-1-3 (PMC4574392; doi:10.1186/2052-6687-1-3)

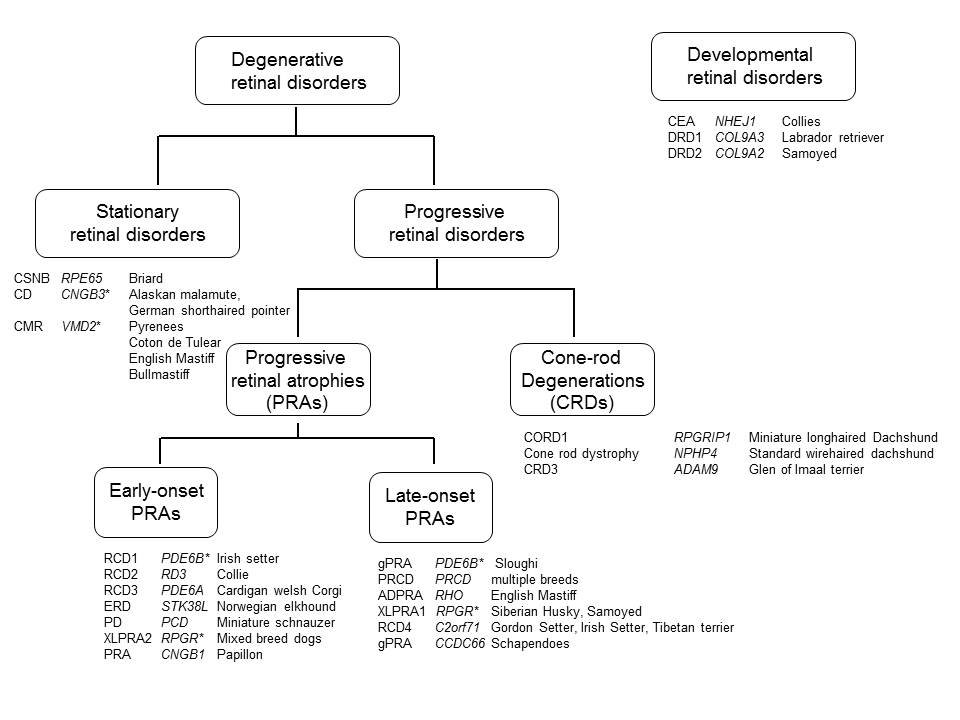

Supplement: Supplementary file 1 — Authors’ original file for figure 1 [file 40575_2013_3_MOESM1_ESM.jpeg]
